# Factors associated with motoric cognitive risk syndrome among low-income older adults in Malaysia

**DOI:** 10.1186/s12889-019-6869-z

**Published:** 2019-06-13

**Authors:** Huijin Lau, Arimi Fitri Mat Ludin, Suzana Shahar, Manal Badrasawi, Brian C. Clark

**Affiliations:** 1 Centre for Healthy Aging and Wellness, Faculty of Health Sciences, Universiti Kebangsaan Malaysia, Jalan Raja Muda Abdul Aziz, 50300 Kuala Lumpur, Malaysia; 2 Department of Applied Chemistry and Applied Biology, College of Applied Sciences, Palestine Polytechnic University, Hebron, West Bank, Palestine; 3 Ohio Musculoskeletal and Neurological Institute (OMNI) and Department of Biomedical Sciences, Ohio University, Athens, OH USA; 4 Program of Biomedical Science, Faculty of Health Sciences, UKM KL Jalan Raja Muda Abd Aziz, Kuala Lumpur, 50300 Malaysia

**Keywords:** Motoric cognitive risk, Low income group, Rural, Obesity, Chronic disease

## Abstract

**Background:**

Motoric cognitive risk (MCR) syndrome is characterized by slow gait and memory complaints that could be used to predict an increased risk of dementia. This study aims to determine the MCR syndrome and its risk factors among low-income (B40) older adults in Malaysia.

**Methods:**

Data from TUA cohort study involving 1366 older adults (aged 60 years and above) categorized as low-income were analysed, for risk of MCR syndrome based on defined criteria. Chi-square analysis and independent *t* test were employed to examine differences in socioeconomic, demographic, chronic diseases and lifestyle factors between MCR and non-MCR groups. Risk factors of MCR syndrome were determined using hierarchical logistic regression.

**Results:**

A total of 3.4% of participants fulfilled the criteria of MCR syndrome. Majority of them were female (74.5%, *p* = 0.001), single/widow/widower/divorced (55.3%, *p* = 0.002), living in rural area (72.3%, *p* = 0.011), older age (72.74 ± 7.08 year old, *p* <  0.001) and had lower years of education (3.26 ± 2.91 years, *p* = 0.001) than non-MCR group. After adjustment for age, gender and years of education, participants living in rural area (Adjusted OR = 2.19, 95% CI = 1.10–4.35, *p* = 0.026), with obesity (Adjusted OR = 3.82, 95% CI = 1.70–8.57, *p* = 0.001), diabetes (Adjusted OR = 2.04, 95% CI = 1.01–4.11, *p* = 0.046), heart disease (Adjusted OR = 2.50, 95% CI = 1.00–6.20, *p* = 0.049) and cancer (Adjusted OR = 6.57, 95% CI = 1.18–36.65, *p* = 0.032) were associated with increased risk of MCR syndrome.

**Conclusion:**

Only 3.4% of older adults from low-income group were identified as having MCR syndrome. Women, those living in rural areas, had obesity, diabetes, heart disease and cancer were more likely to have MCR syndrome. Further investigation on MCR as a predementia syndrome will help in development of preventive strategies and interventions to reduce the growing burden of dementia, especially among individuals with low socioeconomic status.

## Background

Malaysia is fast becoming an aging nation and is expected to reach this status by year 2035 [1]. Aging is accompanied by gradual loss of health and physical strength, especially in the aspects of health and physical strength to the elderly [2]. Other than age, studies showed that health determinants among the elderly include adequate exercise, regular medical check-ups, and the absence of health problems [3]. Older adults’ attitudes towards aging may also affect their physical performance [4]. The prevalence of dementia is expected to rise 3 to 4 times higher in Malaysia as compared to developed countries [1]. Therefore, as a developing country, Malaysia is facing challenges to minimize the healthcare burden and to sustain the medical expenses of the growing number of older population. Abu Bakar et al. [5] found that elderly women were more marginalized and at a disadvantage in socioeconomic aspects of their lives. Therefore, it is essential to increase the accessibility of simple and cost effective dementia risk assessments in order to curtail health care costs.

Gait speed has been accepted as a simple, reliable and valid functional measurement of motor control, strength and gait pattern [6]. Studies suggest that coexistence of cognitive complaints with reduced gait speed may indicate an increased risk of dementia [7–10]. Motoric cognitive risk (MCR) syndrome is a newly defined pre-dementia syndrome characterized by slow gait speed with preserved physical functioning and cognitive complaints without dementia [11]. It can be detected without complex cognitive assessments and is accessible in various clinical settings [12].

A multi-country study reported that the pooled prevalence of MCR syndrome among older adults aged 60 and above was 9.7% [12]. A recent large-scale population study in Japan established the modifiable risk factors associated with MCR [13]. The findings reported that risk factors such as diabetes, depressive symptoms, falls and obesity were associated with increased risk of MCR syndrome.

As yet little is known about the occurrence of MCR syndrome and its risk factors among low-income populations. In Malaysia, the low income or B40 group is the bottom 40% of households with an income of less than RM3, 900 per month. The median and mean household income for this group is RM3, 000 per month and RM2, 848 per month, respectively [14]. The present study aims to determine the prevalence of MCR syndrome and its risk factors among low-income (B40) community dwelling older adults in Malaysia.

## Methods

### Study design and participants

The participants eligible for this study were selected from baseline data of a population-based study focusing on neuroprotective model for healthy longevity (TUA) [15]. The TUA study is described elsewhere (cite reference). This study was conducted in four states of Malaysia (Selangor, Perak, Kelantan and Johor) from November 2014 till September 2015. A total of 1366 multiethnic (Malay, Chinese, Indian) participants were identified as low income (i.e., household income of less than RM 3900 per month) together with other inclusion criteria including: 1) community dwelling older adults aged 60 and above, 2) no psychiatric and mental disorders, included dementia 3) no terminal illnesses and 4) preserved functional ability.

### MCR criteria

MCR syndrome was first proposed by Verghese et al. [11] which is a high-risk clinical syndrome with strong predictive validity for dementia that builds on mild cognitive impairment (MCI) operational definitions [16]. The objective cognitive impairment criterion in MCI is substituted with slow gait in MCR syndrome. Cognitive tests are not needed in diagnosing MCR syndrome. Participants were defined as having MCR syndrome if they meet the criteria as outlined in Table 1.

**Table 1:** Criteria of MCR syndrome

	Criteria of MCR syndrome
1.	Absence of dementia
2.	Subjective memory complaints
3.	Slow gait
4	Preserved activities of daily living (ADL)

Subjects were defined as not having dementia if they scored less than 14 in Mini Mental State Examination (MMSE). A single dichotomous question “Do you feel you have more problems with memory than most?” on Geriatric Depression Scale (GDS) was administered by trained enumerators to elicit the presence of subjective memory complaints. Participants who answered “yes” on this question were defined as having subjective memory complaints. The same question was used to define subjective cognitive complaints in Doi et al.’s [13] study, as well as other cohorts included in the worldwide MCR prevalence study [16]. Preserved activities of daily living including eating/feeding, dressing, bathing and showering, functional mobility, climbing up and down stairs, personal hygiene and grooming, and toilet hygiene, were determined using ADL questionnaire [17]. Gait speed was measured using a 6 m-distance walk on a level floor over time. Participants were instructed to walk back and forth over the marked distance at their usual pace. Slow gait was defined as 1 SD below mean population gait speed [11].

### Potential risk and confounding factors

Potential sociodemography risk factors comprising age, gender, educational years, smoking habit, alcohol consumption, marriage status, and strata status (urban and rural) were determined using a sociodemography questionnaire. Obesity was defined as body mass index (BMI) ≥ 30 kg/m^2^. The presence of chronic diseases (hypertension, diabetes, hypercholesterolemia, arthritis, stroke, cardiovascular disease, chronic obstructive lung disorder, and cancer) was determined using a self-reported medical history questionnaire. Participants were classified as having depressive symptoms if they scored five and above on a 15-items Geriatric Depressive Scale (GDS).

### Statistical analysis

All data were analysed using IBM Statistical Package for Social Science (SPSS) version 22 (IBM Corp., Chicago, IL). Significant value was set at *p* <  0.05. Comparison of characteristics between MCR and non-MCR groups were analysed using chi-squared (χ^2^) tests for categorical variables and independent *t-*test for continuous variables. Hierarchical binary logistic regression was employed to determine the risk factors of MCR syndrome, adjusted for age, gender and educational years. Results were reported as adjusted odd ratio and 95% confidence interval (CI).

## Results

### Prevalence of MCR syndrome

A total of 3.4% of the subjects fulfilled the criteria for MCR syndrome. Women had a higher prevalence of MCR syndrome (74.5%) compared to men (25.5%) (*p* = 0.001). As shown in Table 2, respondents with MCR syndrome were significantly older and had lower educational years than those without MCR syndrome (*p* <  0.001). Majority of them were also living in rural area (*p* = 0.011), unmarried, divorced, widow or widower (*p* = 0.002).

**Table 2:** Comparison of baseline characteristics

Variables	MCR (*n* = 47)	NON-MCR (*n* = 1319)	*p* value
	*n* (%) / Mean ± SD	*n* (%) / Mean ± SD	
Age (years)^a^	72.74 ± 7.08	68.52 ± 5.88	< 0.001^*^
Gender^b^			
Male	12 (25.5)	660 (50.0)	0.001^*^
Female	35 (74.5)	659 (50.0)	
Education (years)^a^	3.26 ± 2.91	5.04 ± 3.81	< 0.001^*^
Marrital status^b^			0.002
Married	21 (44.7)	888 (67.3)	
Single/widow/widower/divorced	26 (55.3)	431 (32.7)	
Smoking Habit^b^			
Smoker	7 (14.9)	239 (18.1)	0.235
Non Smoker	40 (72.1)	1080 (81.9)	
Alcohol Consumption^b^			
Yes	0 (0)	58 (4.4)	0.260
No	47 (3.6)	1261 (95.6)	
Strata Status^b^			0.011^*^
Rural	34 (72.3)	699 (53.0)	
Urban	13 (27.7)	620 (47.0)	
Obesity^b^			0.039
Yes	11 (23.4)	160 (12.1)	
No	36 (76.6)	1159 (87.9)	
GDS^a^	3.64 ± 2.88	3.03 ± 2.33	0.160
*Chronic diseases* ^*b*^			
Hypertension			
Yes	27 (57.4)	675 (51.6)	0.461
No	20 (42.6)	633 (48.4)	
Diabetes			
Yes	17 (36.2)	342 (26.1)	0.132
No	30 (63.8)	966 (73.9)	
Hypercholesterolemia			
Yes	20 (42.6)	530 (40.5)	0.765
No	27 (57.4)	778 (59.5)	
Arthritis			
Yes	12 (25.5)	331 (25.3)	1.000
No	35 (74.5)	977 (74.7)	
Stroke			
Yes	0 (0)	20 (1.5)	0.393
No	47 (100)	1288 (98.5)	
Cardiovascular disease			
Yes	8 (17.0)	116 (8.9)	0.068
No	39 (83.0)	1192 (91.1)	
Chronic obstructive lung disease			
Yes	1 (0.4)	4 (0.3)	0.816
No	228 (99.6)	1183 (99.7)	
Cancer			
Yes	2 (4.3)	10 (0.8)	0.063
No	45 (95.7)	1287 (99.2)	

*GDS* Geriatric Depressive Scale

^a^Independent t-test

^b^Chi-squared test

^*^Significant at *p* < 0.05

### Risk factors of MCR syndrome

Table 3 shows the findings of hierarchical binary logistic regression analysis, adjusted for age, gender and education years. Increasing age (Adjusted OR: 1.13, 95% CI: 1.074–1.197, *p* <  0.001), being female (Adjusted OR: 3.67, 95% CI: 1.485–9.070, *p* = 0.005) and living in rural area (Adjusted OR: 2.19, 95% CI: 1.098–4.348, *p* = 0.026) increased risk of having MCR syndrome. Other factors associated with increased risk of MCR syndrome were obesity (OR: 3.82, 95% CI: 1.699–8.570, *p* = 0.001), diabetes (Adjusted OR: 2.04, 95% CI: 1.013–4.109, *p* = 0.046), cardiovascular disease (Adjusted OR: 2.50, 95% CI: 1.004–6.203, *p* = 0.049), and cancer (Adjusted OR: 6.57, 95% CI: 1.177–36.650, *p* = 0.032).

**Table 3:** Factors that significantly associated with MCR syndrome

Independent variables	Adjusted OR	95% CI		*p* value*
		Lower	Upper	
Age	1.13	1.074	1.197	< 0.001
Gender				
Male				
Female	3.67	1.485	9.070	0.005
Strata Status				
Urban				
Rural	2.19	1.098	4.348	0.026
Obesity				
No				
Yes	3.82	1.699	8.570	0.001
Diabetes				
No				
Yes	2.04	1.013	4.109	0.046
Cardiovascular disease				
No	2.50	1.004	6.203	0.049
Yes				
Cancer				
No				
Yes	6.57	1.177	36.650	0.032

Hierarchical binary logistic regression, Adjusted for age, Gender and educational years, *Significiant at *p* < 0.05

## Discussion

This study showed that the prevalence of MCR syndrome among low- income community dwelling older adults in an Asian country (Malaysia) was 3.4%. This figure is lower than findings from studies conducted in other Asian countries. A meta-analysis showed that the MCR syndrome prevalence among adults from Korea, China, Japan (Kurihara Project) and India (Kerala-Einstein Study), ranged from 10 to 15% [12]. The differences could be due to several factors including age range, sample size and target group [12, 13]. For instance, the highest MCR syndrome prevalence (15%) was reported in an Indian cohort, which enrolled participants with memory complaints only. In addition, the age range of subjects from the present study was 60 to 92 years old, different from that reported in Japan (74 to 95 years old) and Korea (65 to 102 years old). Sample sizes of cohorts in India (*n* = 271), Japan (*n* = 514) and Korea (*n* = 549) were also smaller as compared to the present study [12].

Demographic characteristics of the elderly varied in rural and urban settings in terms of loneliness, lack of financial stability, and emotional strain [18]. Single elderly with poor general health status living in the rural areas were at higher risk of depression [19]. According to Koris et al. [20], majority of the elderly from low- income groups experience castatrophic health expenditure (CHE), with the total direct expenses exceeding 10% of household income. Malaysian elderly in rural areas expressed greater need for health services and experienced more financial hardship than those in urban areas [21]. They still have to be formally employed to maintain their livelihood [19]. Complex neuropsychological testing or neuroimaging services are often limited in rural areas. Therefore, determination of MCR syndrome can be used to predict the risk of developing cognitive impairment and dementia, especially for elderly in rural areas and belonging to the low-income category.

Previous studies showed no significant gender disparities in MCR prevalence [12, 13]. However, in the present study, women had a significantly higher prevalence of MCR compared to men. A study conducted among Malaysian older adults found that women had significantly higher prevalence of frailty (11.8%) than men (5.2%) (*p* <  0.001) [22]. This could be due to the fact that women have lower muscle mass [23] and lose their lean body mass faster than men during the aging process [24] putting them at a higher risk of becoming physically frail.

Similar to the previous studies, participants with MCR were older, less educated, had obesity and diabetes [11, 13]. A meta-analysis on MCR demonstrated that MCR syndrome is significantly associated with cardiovascular disease and its risk factors such as hypertension, diabetes, stroke and obesity [25]. These findings suggest that a vascular mechanism may underlie the pathophysiology of MCR syndrome. Cardiovascular risk factors increased the risk of cerebral ischemia affecting the periventricular white matter [26, 27]. Brain white matter plays an important role in executive function and cognitive processing, as well as control of gait [26, 28]. The effects of diabetes on cognitive decline may relate to macrovascular and microvascular complications. Macrovascular complications such as hyperglycaemia, hyperlidipedimia, hypertension and inflammation may lead to brain structural changes and loss of brain volume [29, 30]. Additionally, microvascular change such as diabetic retinopathy was also associated with lower verbal fluency, mental flexibility and processing speed [31]. Previous studies that have examined the association between arthritis and cognition suggested that arthritis might increase the risk for cognitive impairment [32–34]. Arthritis and cognitive impairment are both associated with factors such as fatigue, pain, depression and increased risk of physical inactivity. However, arthritis was not significantly associated with risk of MCR in the present study.

Previous studies also reported that participants with MCR were more depressed compared to non-MCR group [11, 13]. Our colleagues from the same large-scale population study showed that functional status is one of the predictors that significantly associated with geriatric depressive disorders among Malaysian older adults [35]. Depressive symptoms were also reported highest in Mild Cognitive Impairment (MCI) group [36]. Nevertheless, depressive symptom was not associated with the risk of MCR in the present study. Both MCR and non-MCR groups reported not having any depressive symptom as measured using GDS. This might explain the lack of association of depressive syndrome with MCR.

The strength of this study is that it is one of very few studies investigating MCR among low-income populations in Asia. The limitation of the present study is that the true causal relationships could not be derived as this was a cross sectional study. Nevertheless, the multiple factors associated with MCR syndrome in the present study were in agreement with the risk factors of cognitive impairment and dementia [37]. Future validation studies are needed so that this simple clinical approach can be used to improve dementia risk assessments, develop interventions and preventive measures to optimize cognitive performance of Malaysian elderly.

In conclusion, Malaysian older adults from the low-income (B40) group, especially women living in rural areas, with obesity, diabetes, heart disease and cancer were at a higher risk of MCR syndrome. The cost effective MCR concept can be easily applied in various settings, particularly in rural areas that lack of healthcare facilities, to identify high-risk individuals. Further investigation on MCR as a predementia syndrome will help in development of preventive strategies and interventions to reduce the growing burden of dementia, especially among individuals with low socioeconomic status.

## Abbreviations

ADL: Activities of Daily Living
B40: Bottom 40%
BMI: Body Mass Index
CHE: Castatrophic Eealth expenditure
CI: Confidence Interval
GDS: Gereiatric Depression Scale
MCI: Mild Cognitive Impairment
MCR: Motoric Cognitive Risk
MMSE: Mini Mental State of Examination
OR: Odd Ratio
SD: Standard Deviation
SPSS: Statistical Package for Social Sciences
TUA: Towards Useful Aging
χ^2^: Chi-square
